# How to assess hypercoagulability in heparin-induced thrombocytopenia? Biomarkers of potential value to support therapeutic intensity of non-heparin anticoagulation

**DOI:** 10.1186/s12959-023-00546-8

**Published:** 2023-09-19

**Authors:** Antoine Barocas, Philippe Savard, Audrey Carlo, Thomas Lecompte, Emmanuel de Maistre

**Affiliations:** 1 Haemostasis Unit, CHU, Dijon, France; 2 Diagnostica Stago, Asnières-sur-Seine, France; 3 Vascular Medicine Division, CHU, Nancy, France; 4 Medicine Faculty of Nancy, Lorraine University, Lorraine, France

**Keywords:** Heparin induced thrombocytopenia, Thrombin generation, Fibrin monomers, Procoagulant vesicles, Thrombosis, Anticoagulant treatment

## Abstract

**Background:**

In case of heparin-induced thrombocytopenia (HIT), the switch to a non-heparin anticoagulant is mandatory, at a therapeutic dose. Such a treatment has limitations though, especially for patients with renal and/or hepatic failure. Candidate laboratory tests could detect the more coagulable HIT patients, for whom therapeutic anticoagulation would be the more justified.

**Patients and methods:**

This was a monocentre observational prospective study in which 111 patients with suspected HIT were included. Nineteen were diagnosed with HIT (ELISA and platelet activation assay), among whom 10 were classified as HITT + when a thrombotic event was present at diagnosis or during the first following week. Two plasma prethrombotic biomarkers of in vivo activation of the haemostasis system, procoagulant phospholipids (ProcoagPPL) associated with extracellular vesicles and fibrin monomers (FM test), as well as in vitro thrombin potential (ST Genesia; low picomolar tissue factor) after heparin neutralization (heparinase), were studied. The results were primarily compared between HITT + and HITT- patients.

**Results:**

Those HIT + patients with thrombotic events in acute phase or shortly after (referred as HITT+) had a more coagulable phenotype than HIT + patients without thrombotic events since: (i) clotting times related to plasma procoagulant phospholipids tended to be shorter; (ii) fibrin monomers levels were statistically significantly higher (p = 0.0483); (iii) thrombin potential values were statistically significantly higher (p = 0.0404). Of note, among all patients suspected of suffering from HIT, we did not evidence a hypercoagulable phenotype in patients diagnosed with HIT compared to patients for whom the diagnosis of HIT was ruled out.

**Conclusion:**

The three tests could help identify those HIT patients the most prone to thrombosis.

## Introduction

Heparin induced thrombocytopenia (HIT) is an immune-mediated adverse drug reaction that occurs during heparin administration. It does not expose per se to bleeding risk as other drug-induced thrombocytopenias do, but to thrombotic risk. In the absence of optimal management in the diagnosis and the treatment of HIT, venous and arterial thromboses occur in 50% of cases, which may be limb or life-threatening [1, 2]. This severe side effect has to be timely diagnosed, and urges the use of a non-heparin anticoagulant [3] .

HIT is mostly caused by IgG antibodies directed against platelet factor 4 (PF4)-heparin complexes. As the IgG antibodies bind to platelets through their FcyRIIa receptor [3], they induce platelet activation with consumptive thrombocytopenia and a hypercoagulable state including the production of procoagulant extracellular vesicles, and microparticles, leading, along with activation of other cells in contact of blood plasma, to thrombin generation and thrombotic events [4].

Management of HIT patients requires a change in anticoagulant treatment: switch to parenteral danaparoid, argatroban or fondaparinux [1, 5]. However, those treatments are expensive, more difficult to monitor than heparin (specific laboratory assays being not readily available) and can induce bleeding complications, which can be difficult to manage with no antidote available [3]. In addition, taking into account the risk of life-threatening venous or arterial thrombotic complications, guidelines recommend therapeutic anticoagulation whatever the clinical setting (even if the offending heparin was used with a prophylactic regimen only) [1, 5]. Exposure to a therapeutic dose anticoagulant regimen is not trivial in those HIT patients, often critically ill, with renal and/or hepatic failure [6].

We hypothesized that one or several coagulation assays performed after HIT diagnosis could help detect the patients the most prone to thrombosis thus supporting resorting to a therapeutic dose of a non-heparin anticoagulant. We investigated three laboratory assays: a marker of intense platelet activation with circulating procoagulant phospholipids, associated with extracellular vesicles [7]; a biomarker of thrombin formation in vivo, namely a fibrin monomer assay [8]; and thrombin generation, which measures thrombin potential in vitro [9, 10] whereas the two former are candidate prethrombotic biomarkers. This is a pilot study since to the best of our knowledge no data are available about those markers in the HIT setting.

## Patients, materials and methods

This pilot study was observational and prospective. The protocol was approved by the Ethics Committee : Comité de Protection des Personnes (CPP) Est IV (ID 2018-A00008-47).

### Patients

We screened a total of 201 patients with HIT suspicion between September 2018 and September 2020. Patients were included if their 4T score was > 3, including a platelet count decrease equal or greater than 30% from the initial count and heparin treatment administered for at least 5 days [1].

Of those 201 patients, 83 were not included because they did not meet the inclusion criteria and 13 because not enough plasma was stored after the initial work up. The decision to stop heparin and switch to an alternative anticoagulant is made by the haematologist and the attending clinician, based on the careful examination of the case presentation leading, but not restricted to the 4T score and the results of the first available biological tests results. Sometimes the alternative treatment is promptly started (before blood sampling) indeed ; those cases were not included in the study to avoid possible interference in the three techniques evaluated.

For each included patient, the following clinical and laboratory information was recorded: age, gender, hospital unit (medical, surgical or ICU), nature of anticoagulant treatment and corresponding anti-Xa level, 4T score, platelet count, HIT immunoassay results, heparin-dependent platelet activation results (Table 1).

Laboratory diagnosis of HIT relied on a positive platelet activation test with light transmission aggregometry [11] and/or positive ELISA with an optical density (OD) > 2 (Asserachrom® HPIA Diagnostica Stago, Asnières sur Seine France).

In order to increase the number HIT + patients we added six diagnosed until July 2021. Among the 19 patients diagnosed with HIT (13 over the study period + 6 supplementary patients), ten suffered from a thrombotic event (HITT + patients) present at the diagnosis or during the first week, documented with relevant imaging, such as Doppler ultrasound (US) and computer tomography (CT-scan) for venous thromboembolism. Of note, in case of confirmed HIT diagnosis, systematic Doppler US was systematically performed.

Diagnosis of HIT was based on 4T score, laboratory testing (ELISA anti-FP4 antibodies; platelet activation test with normal PRP and light transmission aggregometry), thrombotic complication, correction of platelet count after shifting to non-heparin anticoagulant treatment (danaparoid or argatroban).

**Table 1:** Characteristics of the study patients

	Total	HIT-	HIT+	Among HIT+	
				HITT-	HITT+
N	111	92	19	9	10
Age (median)	69	69	69	74	60
Sex-ratio (M/F)	60/51	49/43	11/8	5/4	6/4
Nadir platelet count 10^9^/L (median)	70	70	55	52	81
Prothrombin Time ratio (median)	1.27	1.27	1.27	1.44	1.09
Fibrinogen level g/L	4.6	4.4	5.0	5.1	5.0
Intensive Care Unit %	48	45	63	67	60
4T score (median)	4	4	6	5	6

HIT -: patients in whom HIT diagnosis was ruled out

HIT+: patients with HIT diagnosis, with thrombotic complications (HITT+), without thrombotic complications (HITT-)

### Blood collection and preparation

Blood samples were collected at the time of HIT suspicion (initial sampling), into citrate 3.2% tubes (BD Vacutainer, France), 9 volumes of blood for 1 volume of citrate solution. Blood samples were centrifuged at 2500 g for 15 min to obtain platelet poor plasma. Plasma was then aliquoted (1mL) and kept frozen at -80 °C until further analysis. The only anticoagulant treatment potentially present in samples was heparin (unfractionated heparin or low molecular weight heparin).

### Procoagulant phospholipids of circulating extracellular vesicles

Procoagulant phospholipids (ProcoagPPL) associated with extracellular vesicles (phosphatidylserine exposure), formerly known as microparticles, were assessed in plasma samples with a commercially available assay (STA®-Procoag PPL, Diagnostica Stago, France). The assay measures the clotting time initiated with added factor Xa and calcium to the plasma sample mixed with a normal, procoagulant phospholipid-depleted, plasma. The shorter the clotting times, the greater the procoagulant phospholipids plasma levels.

STA®-Procoag PPL is considered to be insensitive to heparin levels up to 1.5 IU anti-Xa/mL (unfractionated heparin) or 2.0 IU anti-Xa/mL (low molecular weight heparin). The kit was used according to manufacturer’s instructions on STA R® Max analyzer (Diagnostica Stago, France).

### Fibrin monomers

Fibrin monomers (FM), also known as soluble fibrin, result from thrombin action on fibrinogen, when fibrinopeptides A are released and fibrin monomers are not polymerized to such an extent that they are no longer soluble [12]. FM levels were measured using an immunoturbidimetric assay (STA®- Liatest® FM, Diagnostica Stago, France) on STA R® Max analyzer according to manufacturer’s instructions. The assay principle is based on immunocapture of FM by a mouse monoclonal anti-human FM antibody coated onto latex microparticles and OD is converted into FM concentration using a dedicated calibration (STA®-Liatest® FM Calibrator, Diagnostica Stago, France).

### Thrombin generation

Thrombin generation (TG) monitors the course of active thrombin over time in a clotting plasma sample [13]. The principle of this assay was already described, with a fluorometric detection of thrombin over time [14–16]. All devices and reagents were from Diagnostica Stago. We studied TG on ST Genesia analyzer using the STG-BleedScreen reagent. The choice of the initiating reagent, the one with the lowest concentration of tissue factor (TF), was aimed at being as sensitive as possible to the expected hypercoagulable states secondary to procoagulant microparticles in HIT. We performed runs of samples from 12 patients, along with one calibration curve (with STG-ThrombiCal), two quality controls measurements (low and normal TG level). The calibration curve was run in parallel with STG-FluoSet enabling calculation of a correction factor for the optical characteristics of each plasma sample as described earlier [20]. Four parameters from the time course of active thrombin i.e. thrombogram were used for further analysis: Lag Time, which corresponds to the time elapsed before the first quantities of thrombin appear (this quantity being equal to 1/6th of the thrombin level at Peak Height); Peak Height, which is the maximum concentration of thrombin; Time to Peak, corresponding to the time to reach the Peak Height; and Endogenous Thrombin Potential (ETP), which corresponds to the area under the TG curve and represents the total thrombin potential of the sample. Results were kept for statistical analysis, as soon as ST Genesia analyzer delivered results with a thrombogram (exclusion if no value was delivered with the message “TG too low”).

TG results were recorded as absolute and normalized against reference plasma STG-RefPlasma BLS [17, 18]. The reference plasma is a lyophilized standard sample to which a correction factor, provided by the manufacturer, is applied to scale results to normal (or 100%) [16]. This normalization step of TG results is meant to smooth for inter-runs and inter-batches variability [19, 20]. However, as our study was run in a single site, using a single batch of reagent and within few runs of tests, we chose to only represent absolute results here. Normalized results, expressed as ratios for lag time and time to peak and as percentages for peak height and ETP, are available as supplementary material.

### Heparin neutralization

Samples sent to the laboratory for HIT diagnosis work up often contained heparin because blood was collected early, as soon as HIT was suspected and TG is very sensitive to presence of heparin in the plasma sample [20, 21]. Consequently, we had to perform the assay after neutralization of heparin. For this, we considered two reagents: polybrene (hexadimethrine bromide, Sigma-Aldrich, France) at concentrations described elsewhere [22] and heparinase (Dade Hepzyme®, Siemens Healthcare Diagnostics, Marburg Germany), using one vial of product for 1mL of plasma, according to manufacturer’s instructions. In preliminary experiments, plasmas from two healthy controls and five non-HIT patients under unfractionated heparin (UFH) and low molecular weight heparin (LMWH) were incubated with polybrene or Dade Hepzyme® at room temperature for 15 min before checking neutralization efficiency as follows. Coagulation tests (APTT, PT) and chromogenic anti-Xa assay (STA®-Liquid Anti-Xa, Diagnostica Stago, France) were performed with a STAR® Max analyzer before and after heparin neutralization. As some residual anti-Xa levels (0.10–0.20 IU/mL) were observed in polybrene-treated samples, we performed the study treating each individual patient’s sample with Dade Hepzyme® before TG study.

### Statistical analysis

Statistical analyses were performed using MedCalc Statistical Software version 17.4.4 (MedCalc Software bvba, Ostend, Belgium). Results of PPL, FM, and TG were compared (i) among HIT + patients, those with or without thrombosis (HITT + and HITT- respectively) primary objective; (ii) between HIT + and HIT- patients.

Statistical significances of differences for each group pair were explored with Mann-Whitney test (cut-off p-value set at 0.05).

## Results

In this study 111 patients were included; 19 patients with established HIT diagnosis (HIT+) and 92 patients for whom HIT was ruled out (HIT-). Among HIT + patients, thrombotic complications occurred in 10 patients: 7 patients with venous thromboembolic event (deep vein thrombosis, pulmonary embolism) and 3 patients with arterial event (2 strokes and 1 occlusion of popliteal artery). Five thrombotic complications were diagnosed on the day of HIT diagnosis. The five other patients presented thrombotic complications a few days after the diagnosis of HIT, despite discontinuation of heparin and introduction of alternative treatment (4 danaparoid and 1 argatroban) at an effective dose (anti-Xa danaparoid: 0.5 and 0.8 U/mL; argatroban level: 0.5–1.5 µg/mL).

All results of measurements with comparison analyses are displayed in Table 2.

**Table 2:** Results of coagulation parameters for each group of patients: prethrombotic biomarkers, and in vitro thrombin generation. Data are presented as median (min-max). The p-values of Mann-Whitney tests were considered significant when lower than 0.05 (marked as bold italic fonts). FM: fibrin monomers, PPL: procoagulant phospholipids, TG: thrombin generation, ETP: endogenous thrombin potential

	HIT- N = 92	HIT+ N = 19	HITT- N = 9	HITT+ N = 10	p-value (Mann-Whitney)	
					HIT- vs. HIT+	HITT- vs. HITT+
**Procoag-PPL** **clotting time (sec)** **N = 105**	51.9 (29.7–148.3)	65.7 (43.6–93.7)	77.3 (43.6–93.7)	56.4 (51.4–62.2)	***0.0027***	0.0956
**FM test** **(µg/mL)** N = 107	5.0 (5.0-150)	6.7 (5.0-150)	5.0 (5.0-150)	96.7 (5.1–150)	0.1790	***0.0483***
**TG Test** N = 108						
** Lag time** **(min)**	5.4 (2.6–15.9)	10.1 (4.7–24.4)	12.5 (5.2–24.4)	8.3 (4.7–12.1)	***0.0001***	0.0637
** Peak Height (nM)**	170.5 (29.2–406.1)	96.6 (8.2-257.2)	96.3 (8.2–192.2)	111.7 (52.9–257.2)	***0.0020***	0.2416
** Time to Peak (min)**	7.8 (4.3–19.5)	12.9 (7.5–32.0)	15.7 (8.2 - 32.0)	11.2 (7.5–16.4)	***< 0.0001***	0.0971
** ETP** **(nM.min)**	1095.1 (299.9–2399.8)	801.9 (116.0–1528.4)	670.8 (116.0–1128.2)	945.7 (563.5–1528.4)	***0.0470***	***0.0404***

In TG test, all patients were studied, but 3 patients with flat thrombogram (message from ST Genesia: “TG too low”) were excluded from the statistical analysis. For logistic reasons FM test and Procoag-PPL clotting assay were not performed in respectively 4 and 6 patients.

### Procoagulant phospholipids of circulating extracellular vesicles

**Fig. 1:**
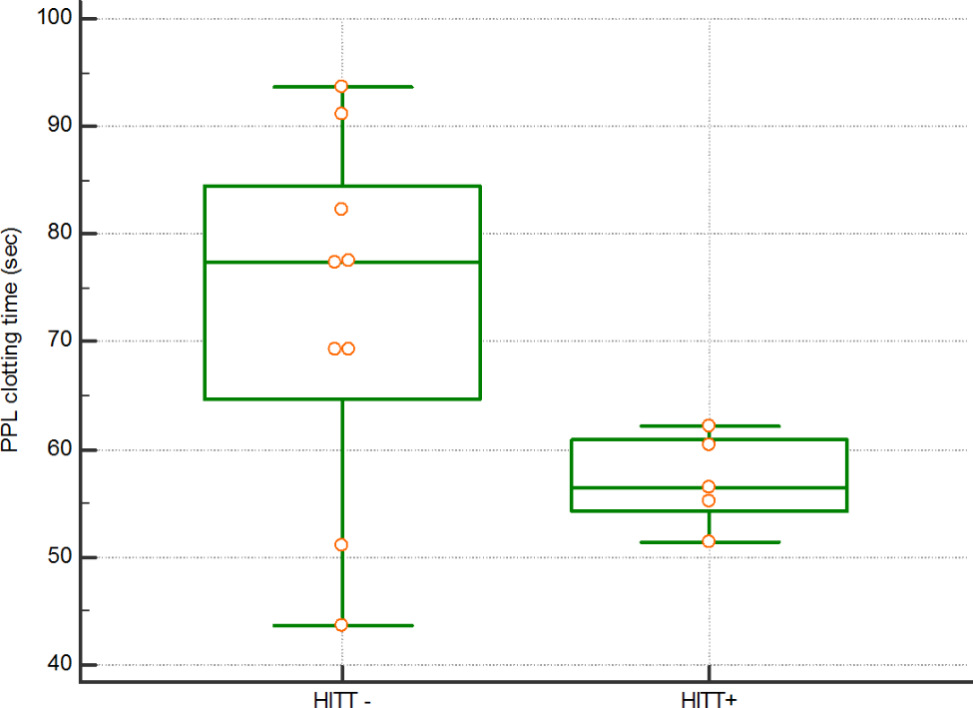
STA®-Procoag PPL clotting times for HIT patients: nine HITT- and five HITT+. Of note five of the additional HIT patients were not tested for procoag PPL for logistic reasons

Clotting times of the procoag-PPL assay were shorter in the HIT- compared to HIT + patients in a statistically significant manner (p = 0.0027, Table 2). Conversely HITT + patients had shorter clotting times than HITT- patients (Fig. 1) though the difference was not statistically significant.

### Fibrin monomers

**Fig. 2:**
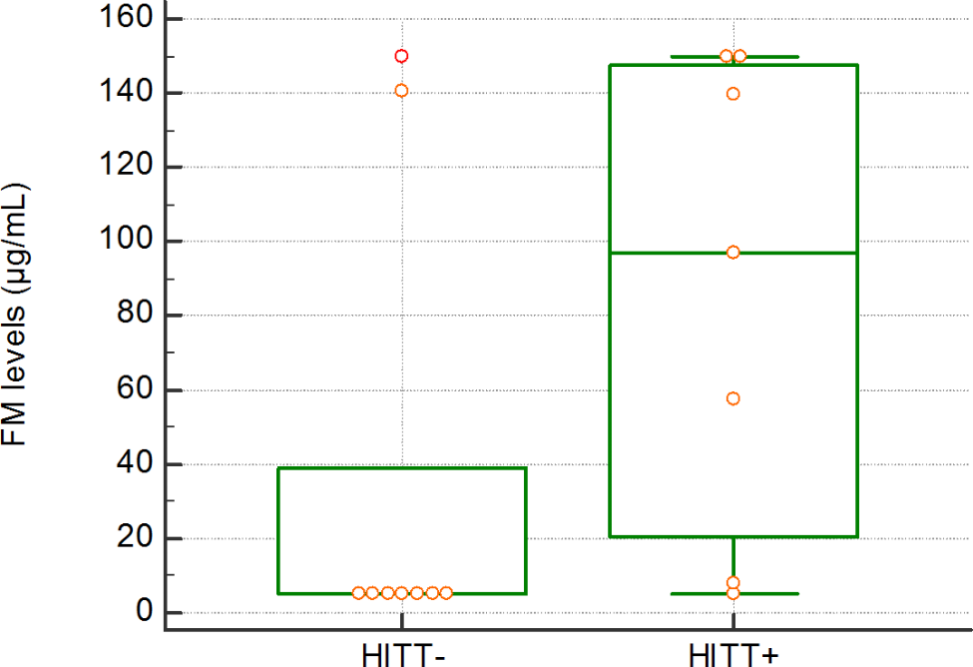
STA®-Liatest® FM results for HIT + patients: nine HITT- and seven HITT + patients. FM levels could not be measured for three of the HITT + patients due to insufficient plasma volume

Results were found to span the overall range of measurement (5.0-150 µg/mL) for all groups of patients. No statistically significant difference was observed between HIT- and HIT + patients. Among HIT patients, FM levels were significantly higher in HITT + compared with HITT- groups (medians: 96.7 µg/mL vs. 5.0 µg/mL; p = 0.0483). The two HITT- patients displayed with high FM levels (Fig. 2), both presented septic shock with disseminated intravascular coagulation (fibrinogen level < 0.8 g/L) after cardiac surgery or TAVI (Transcatheter Aortic Valve Implantation).

### Thrombin generation

TG testing is very sensitive to the presence of heparin, even at low concentration. Hence, we needed to neutralize heparin in the sample, which was achieved with heparinase.

**Fig. 3:**
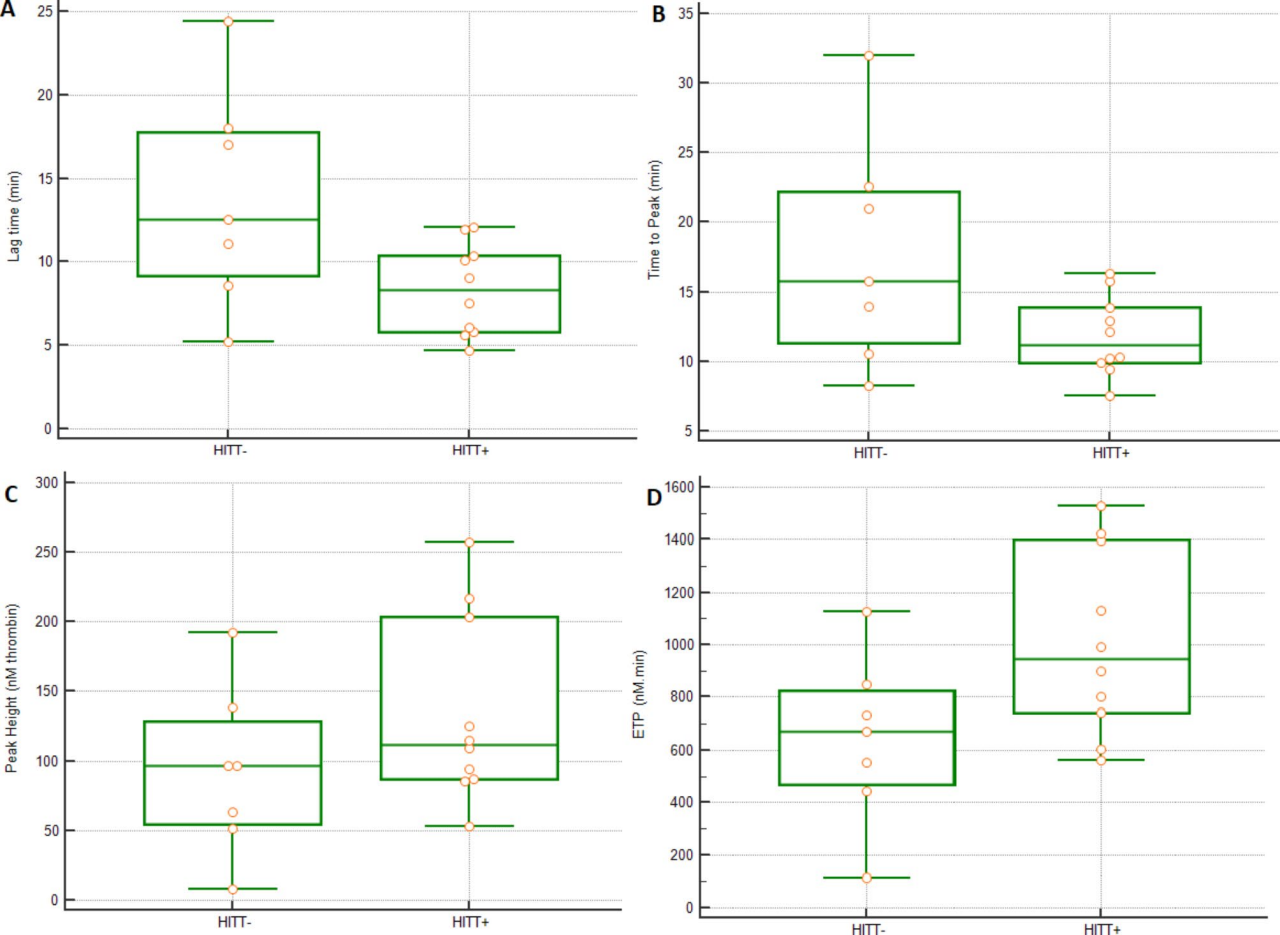
Thrombin generation (ST Genesia), Lag time (A), Time to peak (B), Peak Height (C) and ETP (D) values in HIT + patients: seven HITT- and ten HITT + patients

Among the thrombograms of the included patients, two were very low, likely be due to disseminated intravascular coagulation affecting both patients, hospitalized in intensive care unit. Regarding the patients we could keep for TG data analysis, we unexpectedly observed lower ETP values in HIT + compared with HIT- patients (p = 0.0470***)***. We found however that ETP values were higher in HITT + than in HITT- patients (p = 0.0404). Regarding time-dependent parameters and Peak Height, there were no statistically significant differences between the two groups, though in HITT + patients TG onset occurred earlier, and the Peak Height was higher in comparison to HITT- patients (Fig. 3).

## Discussion

Heparins (UFH and LMWH) are still widely used in prevention and treatment of thrombosis worldwide and HIT is a well-known complication of such a treatment. There is still a strong scientific and medical interest in HIT because the associated major thrombotic risk leading sometimes to limb gangrene and leg amputation or even death [23].

When HIT is diagnosed or strongly suspected, heparin should be stopped and a replacement anticoagulant treatment started, which may vary depending on the country and local habits (danaparoid, argatroban, fondaparinux). In the latest guidelines for the management of HIT [1, 5], a therapeutic dose regimen remains recommended for non-heparin anticoagulants whatever the clinical setting. But the benefit of such dosing is essentially based on one study reported 20 years ago [24], which was compared in a non-randomized manner danaparoid with lepirudin; data collection for danaparoid was retrospective. No differences in outcomes were observed in patients treated with therapeutic doses of danaparoid or lepirudin, but prophylactic dose of danaparoid was reported to be suboptimal (18.6% of thrombotic events vs. 9.4% in therapeutic dose). Those results were not clearly confirmed by any other study, regardless of the anticoagulant (lepirudin, fondaparinux) [25, 26]. The issue of dose intensity concerns danaparoid in particular, the long half-life of which is long (25 h regarding anti-Xa levels) responsible for a delayed steady state, reached after 4–5 days [27]. However the increase in the risk of bleeding with the therapeutic dose should not be overlooked in frail patients, often critically ill.

The evaluation of the thrombotic risk with laboratory tests at the time of the diagnosis of HIT could help in the choice of the intensity of anticoagulation. Three markers were selected for their close relation with thrombin, which is the key enzyme in coagulation cascade and HIT pathogenesis: two prethrombotic markers, Procoag-PPL, FM test and study of thrombin generation (TG). Procoag-PPL levels reflect the intense activation of cells resulting in the formation of circulating extracellular vesicles with exposure of procoagulant phospholipids. FM result from thrombin action on fibrinogen, and are thus a close indicator of circulating thrombin. Finally, TG phenotypes in vitro the potential of the coagulation system. We chose to study TG using the reagent with low concentrations of tissue factor (in the picomolar range) to be sensitive to hypercoagulability. We did not select D-dimers because high levels of D-dimers are common in in-patients, particularly those in the intensive care unit, with multiple causes other than thrombosis [28].

The impact of centrifugation on PPL assay and TG study is debated: the manufacturer recommends a double centrifugation for PPL assay and TG test [29, 30]. Differences between single and double centrifugation were not consistently observed and depended on type of blood collection system (Vacutainer or Monovette) and parameters of TG test [31]. There is a general agreement that the adequate centrifugation protocol is the one that makes the residual platelet count < 10 × 10^9^/L before freezing. This has been regularly checked in daily practice in our laboratory and we undertook a comparison of the two protocols (centrifugation once vs. twice before freezing, 2500 g for 15 min), without observing any difference on TG parameters under the conditions we used.

As expected, HIT patients with thrombosis (HITT+) were more coagulable than HIT patients without thrombosis (HITT-), with higher procoagulant phospholipid levels (trend), significantly higher levels of fibrin monomer (p = 0.0483) and endogenous thrombin generation (p = 0.0404). Among hypercoagulability states, HIT is the only situation that requires therapeutic anticoagulation for all patients diagnosed with this condition, whatever the clinical setting (even if prophylactic regimen could be enough for some), according to the current recommendations. As previously discussed, high doses of anticoagulant may expose patients to the risk of bleeding, particularly in frail ICU patients. The presence or absence of laboratory markers of hypercoagulability at the time of diagnosis of HIT could help in the choice of the anticoagulation regimen. Moreover, part of the thrombotic events were detected within few days after diagnosis of HIT and blood collection for the study of biomarkers, reinforcing the validity of our hypothesis that such laboratory tests could help detect the patients the more prone to those complications, as truly prethrombotic indices.

Surprisingly we did not find in patients with HIT diagnosis (HIT+) indices of a more coagulable state in comparison to patients suspected with HIT, but for whom the diagnosis was eventually ruled out (HIT-). Significantly longer clotting times related to plasma procoagulant phospholipid levels, prolonged lag times and lower thrombin potential with the ST Genesia analyzer were observed in patients diagnosed with HIT.

This observation is at the opposite of our hypothesis but TG, Procoag-PPL and FM results are consistent with one another. Many factors are involved in the TG test and Procog-PPL and we need to continue our investigations. It is possible that the third test (FM test) is more contributory as it is a direct indicator of thrombin formation. As far as we know no study has looked at those three tests in the specific setting of HIT. Patients presenting HIT suspicion are often frail patients with a lot of co-morbidities, and it can be difficult to compare with each other. Of note, patients diagnosed with HIT were critically ill, more often hospitalized in intensive care unit (63% compared to 45% in patients in whom HIT diagnosis was ruled out, p < 0.05) (Table 1).

Another potential explanation for our unexpected data could be the release of tissue factor pathway inhibitor (TFPI) by endothelial cells, induced by heparin therapy. TFPI is bound to glycosaminoglycans on the surface of endothelial cells, with rapid increase in TFPI plasma levels following heparin administration (up to 10 fold after UFH bolus to healthy volunteers [32]. It is known that addition of TFPI to plasma affects TG with prolonged Lag Time and reduced ETP [33]. We hypothesize that the endothelium in HIT patients would release more TFPI, consistent with the multicellular activation taking place while HIT develops [34, 35].

Plasmas samples were incubated with heparinase to neutralize heparin in the samples (heparin levels < 0.1 IU anti-Xa/mL in all samples after treatment) before TG study. We cannot exclude an effect of the neutralizer itself, i.e.; heparinase, on TG test results. Indeed, the impact of heparin neutralization before performing coagulation tests is perhaps not as trivial as the anti-Xa control < 0.1 IU/mL might suggest. In a recent paper [22], Hardy et al. observed an effect of polybrene and also heparinase on TG parameters but tests were performed with plasma samples spiked with heparin. Impact of neutralization agents was different depending on the reagent (STG-ThromboScreen or STG-DrugScreen) and heparin concentration.

In addition, even if heparin neutralization was complete, there likely is an impact of mobilized TFPI from endothelium to circulating blood and we cannot rule out an effect of released TFPI on PPL assay and TG test as already reported on TG at different triggering activities [33].

Our results can be related to those observed by some research groups in the COVID-19 population where, in comparison to TG values in healthy patients, no hint for hypercoagulability was observed in COVID-19 patients and a reduced ETP levels was even observed in severe COVID-19 patients admitted to ICU and treated with heparin, along with a prolonged Lag Time [36].

.

The study presents limitations. First, the small number of HIT patients included advocates a replication with a larger group of patients. Second, the possible residual impact of heparin still present in circulation at the time of blood collection, despite successful heparinase treatment of plasma samples, on PPL and TG results calls for further investigation, e.g. through measurement of TFPI levels. Third, we did not study in parallel TG in a matched healthy population, thus precluding the determination as to whether or not there was an enhanced thrombin potential in HIT patients; and we used just one condition – it might be worthwhile to study TG in presence of thrombomodulin, a condition enabling the detection of some forms of hypercoagulability [37, 38].

## Conclusion

HIT has been considered as one of the most prothrombotic states; thereby therapeutic dose of replacement non-heparin anticoagulation has been repeatedly advocated for all patients suspected of and eventually diagnosed with HIT. Frail patients however, suffering from renal and/or liver failure, are at high risk for bleeding if fully anticoagulated, and thrombocytopenia, which is sometimes marked, cannot be dismissed as another bleeding risk factor. Assessment of the coagulation status of HIT patients could help determine the proper intensity of the replacement anticoagulation. Readily available laboratory tests such as FM test and the study of TG by means of the fully automated ST Genesia device might identify those HIT patients who are the more coagulable and would deserve anticoagulation with a therapeutic dose regimen. A dedicated prospective study is required to validate such an attitude, i.e. that for HIT patients without other risk factors for thrombosis and low values of suitable biomarkers, reduced-intensity non-heparin anticoagulation would be sufficient and safer.

## Data Availability

Available at CHU Dijon Bourgogne from the corresponding author.
